# The Impact of Potent Addictive Substances on Angiogenic Behavior: A Comprehensive Review

**DOI:** 10.2174/1570159X23666240905125037

**Published:** 2024-09-06

**Authors:** Laith Naser Al-Eitan, Saif Zuhair Alahmad, Iliya Yacoub Khair

**Affiliations:** 1 Department of Biotechnology and Genetic Engineering, Jordan University of Science and Technology, Irbid 22110, Jordan

**Keywords:** Alcohol, angiogenesis, cocaine, methamphetamine, opioid, nicotine, cannabinoids

## Abstract

Angiogenesis, the formation of new vasculature from preexisting vasculature, is involved in the development of several diseases as well as various physiological processes. Strict cooperation of proangiogenic and antiangiogenic factors mediates the control of angiogenesis. The fundamental steps in angiogenesis include endothelial cell proliferation, migration, and invasion. Addictive substances, which are considered therapeutic candidates in research and medicine, are classified as natural substances, such as nicotine, or synthetic substances, such as synthetic cannabinoids. Addictive substances have been shown to either enhance or suppress angiogenesis. This review article provides an overview of recent studies concerning the effects of several addictive substances on the process of angiogenesis. Google Scholar and PubMed were used to collect the scientific literature used in this review. The addictive substances addressed in this review are nicotine, opioids such as morphine and heroin, alcohol, cocaine, methamphetamine, and cannabinoids. An accurate assessment of the influence of these substances on the angiogenic process may help to construct a potentially effective therapeutic protocol to control and treat several angiogenesis-related diseases.

## 1 INTRODUCTION

Angiogenesis is defined as the formation and expansion of blood vessels from preexisting vasculature [1]. This physiological process is involved in development and growth, embryogenesis, and wound healing [1] and may also play a role in many diseases. For example, angiogenesis is a crucial step in the malignant progression of cancer. The fundamental steps in angiogenesis include endothelial cell proliferation, migration, and invasion [1, 2]. This process begins in the early embryonic stages known as vasculogenesis, followed by further development of the blood vessels by splitting and sprouting processes. There are two types of angiogenesis: sprouting angiogenesis and intussusceptive angiogenesis. Sprouting angiogenesis is the fundamental mechanism involved in the formation of new vasculature and involves several proangiogenic factors, including Vascular Endothelial Growth Factors (VEGF) and Angiopoietins (ANG), as well as the Wnt and Notch signaling pathways. Intussusceptive angiogenesis, also known as splitting angiogenesis, requires the division or splitting of a larger blood vessel into smaller ones. To accomplish this process, the blood vessel or capillary wall folds into the established lumen of the blood vessel from opposite sides, joining together in order to partition the vessel [1, 2]. The involvement of angiogenesis in several diseases such as cardiovascular disease [3], rheumatoid arthritis [4], liver cirrhosis [5], inflammatory diseases of the synovial joints and the lungs [6], and tumors makes the angiogenic process a vital target for treating these diseases. The enhancement of angiogenesis by specific proangiogenic factors such as VEGF can decrease neurological deficits during stroke recovery [7, 8]. On the other hand, suppression of angiogenesis may help in the management and treatment of tumors [9]. Moreover, Age-related macular degeneration (AMD) is the leading cause of irreversible vision loss in developed countries. The increasing prevalence of late-stage AMD is a significant concern due to the social and economic burdens associated with vision loss in older individuals [10]. Given the importance of angiogenesis and vascular endothelial cell functions in wet AMD, targeting these processes through antiangiogenic therapy is highly desirable. However, unregulated angiogenesis in wet AMD can have devastating consequences. VEGF promotes the formation of weak new vessels beneath the retina, which often rupture and cause bleeding or sub-retinal hemorrhage. Consequently, central vision can be suddenly lost as the retina separates from its support tissue, and scarring of the macula can result from the blood and fluid. This can lead to rapid and severe vision impairment [11]. Considering the role of angiogenesis in wet AMD, inhibiting VEGF presents a logical approach for treatment.

Addiction is a state that affects a person’s brain and behavior and leads to an inability to control the use of legal or illegal drugs. Addiction may begin with the experimental use of a substance for entertainment, followed by more frequent substance abuse [12]. The risk of addiction and the rate at which the patient develops a substance use disorder varies with the drug. Some drugs, such as opioid analgesics, carry higher risks and create dependency more quickly than other substances. Over time, a tolerance to a psychoactive substance develops. As drug use increases, the patient may find it increasingly difficult to discontinue use. Attempts to stop using the drug may cause strong cravings and withdrawal symptoms, which make the patient physically ill [13, 14]. Addictive substances have many characteristics that distinguish them from other substances, and each one has its own effects on the body and brain, depending on the nature of these substances [15]. These substances are classified as natural addictive substances, such as cocaine and cannabis, or synthetic, addictive substances, such as amphetamine and synthetic cannabinoids [16]. Many methods for addiction treatment are under development, such as transcranial direct current stimulation (tDCS), which was observed to reduce craving in addiction patients in the short term [17]. On the other hand, repetitive transcranial magnetic stimulation (rTMS) has not shown a significant reduction in craving and consumption in patients [18]. This review aims to summarize the impact of several addictive substances on the process of angiogenesis, which have been extensively studied for their potential therapeutic use for the control and treatment of angiogenesis-related diseases. For instance, the role of angiogenesis in tumor development is significant, as the capacity of tumors to stimulate new blood vessel growth is linked to tumor recurrence and metastasis [19]. Angiogenesis is a crucial mechanism in cancer metastasis and progression, leading to suggestions that inhibiting this process could be a potential strategy for cancer therapy [20-22]. The substances addressed herein are some of the most addictive, and they are nicotine, opioids such as morphine and heroin, alcohol, cocaine, methamphetamine, Benzodiazepines, and cannabinoids.

## 2 SEARCH METHODOLOGY

To comprehensively review the association between addictive substances and the progression of angiogenesis, we conducted a systematic literature search using PubMed and Google Scholar, employing terms such as “Angiogenesis,” “Cannabis,” “Addictive substances,” “Nicotine,” “Morphine,” “Alcohol,” “Cocaine,” “Methamphetamine,” “Synthetic Cannabinoids,” “Heroin” and “psychosis.” We included peer-reviewed articles, reviews, and meta-analyses published in English from 2000 onwards, focusing on studies involving human subjects as well as *in vitro* assays on cultured cell lines. Data from 139 selected studies were synthesized narratively, highlighting key themes such as epidemiological evidence, genetic factors, and biological mechanisms to provide a comprehensive overview of current research on this topic.

## 3 THE MECHANISM OF ANGIOGENESIS

Blood vessels are a component of the cardiovascular system responsible for transporting nutrients, wastes, gases, and proteins through blood flow. There are five types of blood vessels, including arteries and arterioles, which carry blood rich in oxygen and nutrients away from the heart, capillaries where diffusion of gases and compounds between the body's tissue and blood occurs; and venules and veins, which carry blood rich in carbon dioxide back towards the heart [23, 24].

**Fig. (1):**
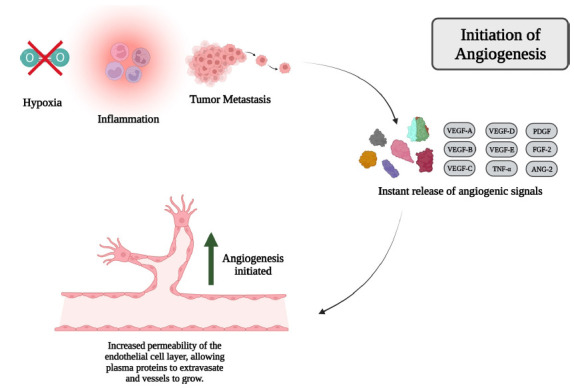
An overview of the mechanism of angiogenesis.

Endothelial cells under normal and healthy conditions are called quiescent endothelial cells (QECs). QECs are composed of a single layer of phalanx endothelial cells that are interconnected by tight junctions such as vascular endothelial cadherin (VE-cadherin) and claudins [1]. QECs are enclosed by pericytes, which prevent the release of proangiogenic factors and restrain the proliferation of the endothelial cells. Moreover, QECs contain oxygen sensors that regulate the concentration of oxygen in the cells and hypoxia-inducible factors (HIFs), which modify the structure of the blood vessels and optimize blood flow in response to hypoxia [1, 25]. Normal or pathological conditions such as inflammation, hypoxia, and cancer metastasis release angiogenic signals *via* VEGF, Platelet-derived Growth Factor (PDGF), Fibroblast Growth Factor-2 (FGF-2), Insulin-like Growth Factors (IGFs), Angiopoietin-2 (ANG-2), Tumor Necrosis Factor (TNF), and chemokines such as Interleukin-6 (IL-6) [26]. The release of these factors leads to the remodeling of the extracellular matrix by proteolytic degradation *via* matrix metalloproteinases (MMPs), thus detaching the pericytes from the vessel wall *via* ANG-2. Subsequently, the tight junctions between QECs lose their function, and the new vessel starts to form. The primary group of proangiogenic molecules is the VEGF family, which is comprised of five members: VEGF-A, VEGF-B, VEGF-C, VEGF-D, and placenta growth factor (PGF) (Fig. **1**). VEGF-A, the primary member of the VEGF family, induces both normal and pathological angiogenesis *via* VEGF receptor-2 (VEGFR-2) signaling pathways. VEGF-A is responsible for the increased permeability of the endothelial cell layer, allowing plasma proteins to extravasate [1, 27].

As a result of sensing the proangiogenic molecules, one endothelial cell is selected and transformed into a tip endothelial cell, which navigates through the extracellular matrix through adhesion and de-adhesion processes that lead to cell migration [1]. VEGF receptors, Notch signaling ligands JAGGED1 and DLL4, and Neuropilins (NRPs) are involved in selecting the tip endothelial cells and preventing other endothelial cells from sensing proangiogenic molecules and subsequently transforming into tip endothelial cells. Tip cell neighbors assume secondary roles as stalk cells that break off to elongate the stalk *via* NOTCH signaling, NOTCH-regulated ankyrin repeat protein (NRARP), FGFs and PGF signaling, and Wnt signaling pathways. Stalk endothelial cells form the lumen with numerous signaling factors, such as VEGF, VE-cadherin, hedgehog, and sialomucins [1, 28]. Subsequently, stalk cells elongate and proliferate, forming a new blood vessel [28]. The blood vessels must become stable and mature in order to become functional. Accordingly, plasminogen activator inhibitor-1 (PAI-1) and tissue inhibitors of metalloproteinases (TIMPs) trigger the re-formation of the basement membrane and intercellular junctions to achieve optimal blood flow. The pericyte layer is re-established *via* NOTCH, ANG-1, ephrin-B2, PDGF-B, and transforming growth factor-β (TGF-β) signaling, and the endothelial cells switch into the quiescent phalanx state [29, 30].

## 4 IMPACT OF ADDICTIVE SUBSTANCES ON ANGIOGENESIS

### 4.1 Nicotine

Nicotine is extracted from the leaves of Nicotiana tabacum. Nicotine can be consumed by tobacco smoking, sniffing, or chewing [31]. Several studies have investigated the potential therapeutic role of nicotine in treating a variety of diseases, such as Alzheimer’s and Parkinson’s disease [32, 33]. On the other hand, nicotine is described as a highly addictive substance that has serious consequences if abused. Nicotine product consumption is a serious public health issue, with smoking being the primary cause of avoidable death in the United States [34]. Cigarette smoking is the leading risk factor for developing cardiovascular disease (CVD), and smokers are two to four times more likely than nonsmokers to develop CVD [35].

Furthermore, there is substantial evidence to substantiate the notion that a favorable association exists between the act of smoking cigarettes and the probability of acquiring lung cancer. Lung cancer stands as the foremost cause of cancer-related fatalities in both North America and other industrialized nations [36]. Ceasing smoking activities may potentially diminish the chances of developing secondary primary lung cancer (SPLC) [37]. The overwhelming majority (85%) of lung cancer cases can be attributed to tobacco usage, wherein the duration of smoking holds a more significant influence than the quantity consumed. Additionally, exposure to secondhand smoke escalates the risk of lung cancer and accounts for approximately a quarter of all lung cancer incidents among non-smokers in France [38]. Although combustible cigarette smoking has dropped in the past decade, nicotine exposure has increased significantly due to the growing use of tobacco-free, electronic nicotine delivery systems (*e.g*., vaping and electronic cigarettes), particularly among teenagers and young adults [39]. Nicotine, the predominant psychoactive ingredient of tobacco, has diverse effects on specific brain regions at different stages of development [40]. Thus, nicotine is not only dangerous to adult health but also has neurological effects on fetuses, neonates, children, and teenagers [41].

Alpha 7 nicotinic acetylcholine receptor (alpha7 nAChR) is widely expressed in the central and peripheral nervous systems and is also found in several non-neuronal tissues, such as endothelial cells (ECs). alpha7 nAChR is involved in regulating cellular function in ECs and capillary formation in myocardial infarction, which are the essential steps of angiogenesis. Therefore, alpha7 nAChR on ECs may be a new endothelium target for revascularization in therapeutic angiogenesis of ischemic heart disease [42]. Nicotine-induced angiogenesis required nAChR function and was associated with the upregulation of MMP-2 and -9 in HRMECs. The α7-nAChR is vital for the proangiogenic activity of nicotine. The α7-nAChRs expressed on HRMECs upregulate levels of MMP-2 and -9, which stimulate retinal angiogenesis in primary human retinal microvascular endothelial cells (HRMECs) [43]. Recent studies have shown that nicotine can enhance angiogenesis and arteriogenesis in several experimental systems and animal models. The pro-angiogenic activity of nicotine is mediated by nicotinic acetylcholine receptors, which have been found to be expressed on several types of cells in the vasculature, like endothelial cells, smooth muscle cells, and immune cells [44].

Nicotine demonstrates a significant effect on endothelial cells. Several studies have demonstrated that nicotine increases the number of viable endothelial cells [45], promotes the formation of the capillary network *in vitro*, increases the proliferation rate of endothelial cells, decreases endothelial cell apoptosis, and thus enhances the capacity for angiogenesis [45]. The effect of nicotine is mediated through the augmented release of proangiogenic growth factors such as VEGF and FGF [45, 46]. This finding was confirmed by using α7-nAChR antagonists to block the effects of VEGF or FGF and inhibit the migration and proliferation of endothelial cells. The angiogenic effect of nicotine was also shown to be related to nitric oxide synthesis. Nicotine increases the synthesis of and response to nitric oxide [46]. α-bungarotoxin and Mecamylamine, both α7-nAChR antagonists, have shown a complete and reversible suppression of endothelial tubule formation *in vitro* [47]. Moreover, a high concentration of nicotine could induce morphological alterations in the human umbilical vein endothelial cells (HUVECs), such as cell lengthening and intracellular vacuolization [48]. Pittilo *et al*. (1990) proposed that these morphological alterations in endothelial cells correlate with functional modifications. It is assumed that these alterations are related to a direct cytotoxicity effect at high concentrations of nicotine [49].

Furthermore, nicotine cytotoxicity inhibits endothelial cell proliferation. Systemic exposure to nicotine increases the number and transmigration rate of endothelial progenitor cells (EPCs) in the spleen and bone marrow. This elevation of EPCs is correlated with a pronounced increase in angiogenesis in ischemic tissue [50]. The proangiogenic effect of nicotine was observed in pathological conditions such as ischemia, atherosclerosis, and inflammation [51]. Accelerated growth of tumors and atherosclerotic lesions were correlated with nicotine-enhanced vascularization [46]. Consequently, targeting α7-nAChR to treat angiogenesis-related diseases could be an effective therapeutic approach. For instance, Kathleen *et al*. reported that the α7-nAChR antagonist MG624 showed antiangiogenic activity in small-cell lung cancer (SCLC). Through studies conducted in nude mice and chicken chorioallantoic membrane models of angiogenesis, Brown *et al*. (2012) demonstrated that MG624 strongly suppresses primary human microvascular endothelial cell proliferation in the lung. The anti-angiogenic effect of MG624 was mediated through a decrease in nicotine-induced FGF levels in the microvascular endothelial cells [50].

### 4.2 Opioids

Opioids are a diverse group of drugs that include natural compounds derived from the opium poppy, such as morphine and codeine, as well as synthetic and semi-synthetic compounds. While some opioids, such as fentanyl derivatives, methadone, meperidine, and levorphanol, are used for medicinal purposes, there are also over 70 compounds classified as new/novel psychoactive substances (NPS) that are used recreationally and pose significant risks [52]. Typical side effects of taking opioids include vomiting, sedation, constipation, nausea, dizziness, respiratory depression, and physical dependence [53].

Despite the frequent use of opioids as therapeutic drugs for pain relief, these opioids are highly addictive, making individuals more likely to take them frequently for recreational purposes [54-57]. Opioid abuse usually results in gradual increases in drug doses that are higher than the usual exploratory or analgesic range, which have many side effects, such as respiratory depression, inhibition of gastrointestinal activity, and sedation Whereas, the short-term therapeutic use of opioids, it does not cause any major health problems [54-57].

Morphine, for example, is an alkaloid of opium that acts as a potent analgesic used to manage chronic and severe pain [58]. Morphine abuse leads to the risk of developing morphine use disorder and physical dependence, and therefore, its use should be limited and only available on prescription [59]. Different modes of morphine delivery may be used and include intravenous, subcutaneous, and oral administration, among others. Morphine is an agonist ligand for three types of Opioid receptors: κ-opioid receptors (KOR), μ-opioid receptors (MOR), and δ-opioid receptors (DOR). These receptors are widely expressed in the CNS and PNS [60].

The opioid effects on both angiogenesis and cancer are highly controversial, as their pro and anti-effects on neovascularization and tumor growth have been documented [61, 62]. Codeine, oxycodone, and fentanyl are agonists for μ-opioid receptors, even though they have different affinities for binding to and activating the δ and κ receptors [63]. Previous study shows that fentanyl promotes wound healing and hence stimulates angiogenesis in diabetic rats [64]. Another investigation using HUVEC revealed that fentanyl exhibits strong pro-angiogenic properties, and oxycodone has a moderate pro-angiogenic effect. In contrast, codeine has no impact on the angiogenesis of endothelial cells [65]. Studies on the effect of morphine on tumor-related angiogenesis are inconsistent and contradictory. Morphine enhances angiogenesis under oxidative stress conditions and serum depletion [66]. It also inhibits the angiogenesis-related to tumor growth in mice [67]. Morphine is considered a potent proangiogenic stimulator [65] and has shown a proangiogenic effect in recurrent postoperative breast cancer and increased the proliferation of cancer cells [68, 69]. Morphine has also enhanced the metastasis of breast cancer cells in a dormant state by activating the PI3K-c-Myc signaling pathway [68]. On the contrary, several studies have found that morphine could trigger apoptosis in cancer cells, such as breast and lung cancers [70, 71]. Researchers have found that morphine stimulated angiogenesis in both an *in vitro* experiment with the MDA-MB-231 human breast cancer cell line and an *in vivo* experiment with a heterotopic mouse model. Their results demonstrated that morphine increased proliferation and decreased apoptosis in the MDA-MB-231 cells. Similarly, the *in vivo* study using nude mice model showed that tumor size was increased and neoangiogenesis was enhanced in treated groups compared with untreated groups [72]. Recent study demonstrated that morphine enhances cell, migration, proliferation, and tube formation in HUVECs stimulated by Conditioned medium of the BEL-7402 and HCC-LM3 cell lines [73]. In contrast with studies showing morphine’s proangiogenic effects, several experiments have demonstrated that morphine may also have an antiangiogenic effect, which affects tumor progression. Morphine has been shown to impair leukocyte trans-endothelial migration and decrease tumor-related angiogenesis in both *in vitro* and *in vivo* models [71]. Previous studies conducted on mice suggest that angiogenesis is suppressed by morphine both directly and indirectly throughout the wound-healing process, and macrophages are involved in this suppression [74]. Additionally, in a murine Lewis lung carcinoma cell culture model, morphine suppresses tumor growth and angiogenesis when compared with negative controls. Vessel length, vessel density, and vessel branching were significantly decreased after morphine treatment. Furthermore, MOR knockout mice and the coadministration of morphine with naltrexone (opioid receptor antagonist) have revealed the importance of the opioid receptor in mediating morphine’s inhibitory effect on tumor growth. This inhibitory effect is mediated by blocking the translocation of hypoxia-inducible transcription factor 1 (HIF-1) upon its induction by hypoxia, resulting in a decrease in the release of VEGF [67]. Furthermore, Lam *et al*. (2008) have shown that a high dose of morphine is correlated with elevation of local and systemic oxidative stress, impairment of endothelial progenitor cell recruitment, and suppression of angiogenesis capacity, and therefore impairment of wound healing. Attenuation of the physiological activity of nitric oxide released from endothelial cells led to impairment of vascular endothelial functions as a response to high-dose morphine. This attenuation could result from the chemical antagonism between superoxide anions after treatment with high-dose morphine [75]. These conflicting findings are likely to be associated with different experimental conditions such as morphine concentration, administration time, and tumor cell line. Indeed, *in vivo,* and *in vitro* studies have shown that the proangiogenic effect of morphine is associated with minimal or single daily doses of morphine, while the antiangiogenic effect of morphine is related to chronic high doses of morphine [76].

### 4.3 Alcohol

Alcohol is an organic compound carrying a hydroxyl group (-OH) and is described by the chemical formula CnH2n+1OH. Alcohol was widely used as the primary pharmaceutical treatment for anxiety and insomnia in biblical times [77]. For centuries, the psychoactive alcohol ethanol has been used for recreational purposes and remains one of the most tremendous burdens on the health of society. Alcohol consumption increases the risk of developing alcoholism. It may increase the risk of developing alcoholic pancreatitis, alcoholic liver disease, and cancer, as alcohol is known to be a leading cause [78, 79]. Several studies have proven that ethanol increases the risk of the progression and development of liver, breast, prostate, upper digestive tract, pancreatic, and colorectal cancers [80-84]. In a study by Tan *et al*. (2007), chronic alcohol consumption was shown to stimulate angiogenesis and the progression of melanoma growth in an *in vivo* model [85].

Similarly, Lu *et al*. (2014) demonstrated that alcohol intake accelerated angiogenesis in breast cancer cells as well as tumor growth by increasing the release of VEGF from cancer cells both *in vivo* and *in vitro*. The results were confirmed by blocking VEGF signaling using SU5416, which inhibited tumor angiogenesis [86]. Monocyte chemoattractant protein-1 (MCP-1) is a pro-inflammatory chemokine that has a crucial role in the development and aggressiveness of breast cancer. An overexpression of MCP-1 and C-C chemokine receptor type 2 (CCR2) was reported in breast cancer cells *in vivo* and *in vitro*.

Furthermore, MCP-1 stimulated alcohol-induced angiogenesis, thus increasing breast cancer progression [87]. Ethanol has also been shown to accelerate wound recovery by stimulating the release of VEGF and epidermal growth factor (EGF) from the granulation tissue, which leads to enhanced angiogenesis in both *in vitro* and *in vivo* models [88].

Conversely, in an *in vivo* and *in vitro* study, a single ethanol dose (1.4 g/kg) was associated with a reduction in the rate of angiogenesis and collagen content in the wounds of ethanol-treated mice compared to control mice, and thus with a delay in wound recovery [89]. This antiangiogenic effect was observed despite the presence of sufficient levels of VEGF, indicating that ethanol could have a direct effect on endothelial cell signaling [89]. Furthermore, ethanol administration decreased the expression and phosphorylation of VEGF receptor-2 [90]. Angiogenesis osteogenesis coupling is a spatiotemporal interaction between ossified tissues and blood vessels, resulting in bone formation [91-93]. Yu *et al*. reported that ethanol significantly decreased the osteogenesis and proliferation of bone mesenchymal stem cells *in vitro*, as well as the vasculogenesis and proliferation of HUVECs by upregulating HDAC3 [94].

### 4.4 Cocaine

Cocaine is a potent addictive compound derived from the plant family Erythroxylaceae, which has historically had limited medical uses, including as a local anesthetic [95]. Cocaine addiction may develop after even a short period of abuse. Moreover, cocaine use is associated with an increased risk of sudden cardiac death and an elevated risk of the development of several diseases, including myocardial infarction, respiratory system disorders, and ischemic attacks [95, 96]. The administration of cocaine may occur through several methods, including snorting (nasal inhalation), smoking, oral ingestion, and injection. The analgesic effect of cocaine is mediated by blocking voltage-gated sodium channels in neuronal membranes, which stops the conduction of nerve impulses conduction, thus resulting in loss of sensation [97]. Chronic cocaine uptake has shown a proangiogenic effect mediated by an increase in the expression of VEGF and HIF-1α [98].

Moreover, a significant increase in cerebral microvascular density in the cortical regions of the brain was observed in response to neuroadaptations resulting from cocaine use. The activation of the HIF-VEGF signaling pathway leads to enhanced brain angiogenesis and concomitantly to restored oxygen supply after ischemic attacks [98]. Furthermore, cocaine consumption is related to endothelial dysfunction, which may lead to severe disease. For example, chronic cocaine consumption alters endothelial function towards a pro-thrombotic condition, which may lead to the development of an ischemic vascular disorder in cocaine abusers [99].

### 4.5 Methamphetamine

Methamphetamine is a highly addictive stimulant drug that influences the CNS. It can be consumed nasally (snorting), orally, injected, or smoked, resulting in short- and long-term health repercussions for addicted users [100]. Methamphetamine abuse is a major global public health issue with severe psychological and medical implications such as dependence, psychosis, overdose/death, and socioeconomic, cognitive, and legal consequences [100]. Recreational use of methamphetamine may lead to deficits in cognitive function (including memory loss) and decision-making, neurotoxicity, and risk of methamphetamine-use disorders [101]. Notably, methamphetamine exposure causes toxicity in the human brain microvascular endothelial cells, which affects the blood-brain barrier (BBB) [102]. Nicotine and methamphetamine were found to increase the levels of amyloid-beta, ubiquitin C-terminal hydrolase L1 (UCHL1), and tau protein in the brain microvascular endothelial cells by upregulating α7-nAChR both *in vivo* and *in vitro* models, including in mouse brains and cerebrospinal fluid. The role of α7-nAChR-induction by nicotine and methamphetamine was validated through the use of the α7-nAChR antagonist methyllycaconitine [103]. In an *in vitro* model, a high concentration (2.5 mM) of methamphetamine increases dehydrogenase release and reduces the proliferation of bovine brain microvessel endothelial cells. Furthermore, morphological changes were observed in the endothelial cells at different methamphetamine concentrations; vacuole formation was induced at 1 mM, whereas a complete disruption of the endothelial monolayer was induced at 2.5 mM [104]. Methamphetamine induces inflammatory and oxidative stress, which stimulates the release of extracellular vesicles called microparticles from the cell surface. Microparticle levels change during pathological situations, indicating a possible biomarker role for these vesicles. Methamphetamine was demonstrated to trigger endothelial injury and dysfunction mediated by the release of endothelial-derived microparticles [105]. A recent study conducted *in vivo* proved that methamphetamine-stimulated retinal angiogenesis significantly increased vascular density and numbers of arterioles with accompanying increases in VEGF, HIF-1, and hypoxia [106].

### 4.6 Cannabinoids

Cannabis is considered the third most popular controlled substance globally, following alcohol and tobacco. In 2018, the United Nations evaluated that 3.9% of the world's adult population, with an estimated 192 million people, had taken cannabis in the year before [107]. Cannabis use disorder (CUD) is generally described as the inability to stop using cannabis despite its negative consequences, whether psychological or physical harm [108]. According to the latest global estimates, 22.1 million people were diagnosed with CUD in 2016 (approximately 289.7 cases per 100,000 individuals) [109]. CUD is significantly higher in individuals suffering from mental illnesses, such as personality disorders, anxiety, and mood disorders, post-traumatic stress disorder, and schizophrenia, than in the overall population [110, 111]. The term “cannabis” refers to the cannabis plant, its derivatives, or its extracts, which bind to the cannabinoid receptor CBR-1 in the brain to produce the psychoactive effects (the “high”) desired by cannabis users [112, 113]. It may cause users to have a “high” desire to use it frequently, which may develop into CUD [114]. Additionally, those who have CUD are more likely to suffer from bronchitis, psychosis, and poor mental health [107]. Natural cannabinoids and synthetic cannabinoids induce different symptomatic pictures and outcomes in psychotic patients in comparison to the psychotic symptoms not associated with substance abuse [115].

Cannabis contains a variety of cannabinoids that make up the *indica* plant or *cannabis sativa*, including tetrahydrocannabinol (THC) and cannabidiol (CBD), which have served as the main focus for clinical interests and research [116]. The average THC content in dried cannabis is around 15%, but some strains contain significantly higher levels, with an average content of up to 30%. Cannabis's psychoactive effects are generated mainly by the partial agonist actions of THC on cannabinoid receptor 1, which produces the “high” that is felt by users [117]. On the other hand, early studies have shown that CBD has therapeutic potential such as anxiolytic, anti-craving pro-cognitive, antipsychotic, and neuroprotective effects, as it seems to have divergent and perhaps antagonistic pharmacological action in comparison to THC [118]. Clinical manifestations range from relaxation and euphoria to psychosis and panic anxiety because cannabis products have a complicated pharmacological structure, in addition to the broad distribution of cannabinoid receptors across the brain that regulate a range of neurotransmitters [118, 119].

Cannabinoids are lipid-soluble ligands for cannabinoid receptors on the cell surface. This class of chemicals is classified into three types: endocannabinoids, phytocannabinoids, and synthetic cannabinoids. Phytocannabinoids are produced naturally from Cannabis plants. Approximately 100 phytocannabinoids have been identified, with THC as the primary psychoactive component [120, 121].

*In vivo* and *in vitro* studies demonstrated that chronic intermittent hypoxia (CIH) enhanced the expression of CBR1 and CBR2, which stimulated angiogenesis, tumor growth, and lung metastasis *via* activating IGF1R/AKT/ GSK-3b signaling pathways in hypoxic breast cancer [122]. On the other hand, the knockdown of CBR1 and CBR2 in CIH conditions can block invasion and migration in T47D and MCF7 cell lines by inactivating the same pathways. Additionally, CBR1 and CBR2 silencing *in vivo* prevented the malignancy of breast cancer under normoxia or CIH circumstances [122]. The importance of endocannabinoid system receptors in the angiogenic process makes them a vital target in treating several diseases, including cancer [123]. Over-expression of cannabinoid receptors and endocannabinoid levels in cancer cells has been observed in various tumors. Pisanti *et al*. (2011) have reported that the inactivation of CBR-1 leads to suppression of FGF-induced endothelial cell migration, proliferation, and capillary-like tube formation through pro-survival and migratory pathways [124]. Moreover, many synthetic cannabinoids such as XLR-11, (R)-5-fluoro ADBAB-CHMINACA, 5-fluoro ABICA, 5-fluoro MDMB PICA, and MDMB FUBINACA have been found to increase the viability of human brain microvascular endothelial cells and enhance angiogenesis capacity *in vitro* [125-131]. It is crucial to consider that these synthetic cannabinoids are highly toxic and are classified as new psychoactive substances. They are often misused as substitutes for marijuana, posing significant health risks due to their high potency and unpredictable effects [132]. Blocking both cannabinoid receptors decreased inflammatory angiogenesis, suggesting an essential role for these receptors in the blood vessel formation process [133]. On the other hand, the activation of cannabinoid receptors may trigger antiangiogenic signaling. For example, cannabinoid receptor activation suppresses tumor vascularization *via* down-regulation of several proangiogenic factors such as VEGF, ANG-2, and PGF [134, 135]. Furthermore, Blلzquez *et al*. (2003) demonstrated two mechanisms that mediate the antiangiogenic signals after local administration of non-psychoactive cannabinoids, WIN 55,212-2 and JWH-133, *in vitro* and *in vivo*: first, lowered expression of matrix metalloproteinase-2 and proangiogenic factors such as VEGF and ANG-2 in tumors, and second, direct suppression of the migration and survival of vascular endothelial cells [136]. Further studies conducted *in vitro* and *in vivo* showed that WIN 55,212-2 reduced proliferation, tube formation, and angiogenesis, reduced proliferation and angiogenesis, and promoted apoptosis by changing the protein kinase signaling cascade [136, 137]. Many recent studies by Al-Eitan *et al*. investigated the effect of several synthetic cannabinoids (XLR-11, 5-fluoro ADB, 5-fluoro MDMB PICA, EMB-FUBINACA, MDMB FUBINACA) on the gene and protein expression of VEGF, ANG-1, and ANG-2 in human brain endothelial cells. The mentioned drugs caused the expression levels of the angiogenic proteins to increase significantly, alongside the noticeable increase in the cell’s capacity to migrate and form tube-like structures [126-131]. Further study revealed that HU-331 (cannabidiol hydroxyquinone), a novel CB anticancer quinine, suppresses angiogenesis *via* directly triggering apoptosis of vascular ECs without affecting the production of cytokines that stimulate and inhibit angiogenesis and their receptors [138].

## 5 CONCLUSION AND FUTURE DIRECTIONS

**Fig. (2):**
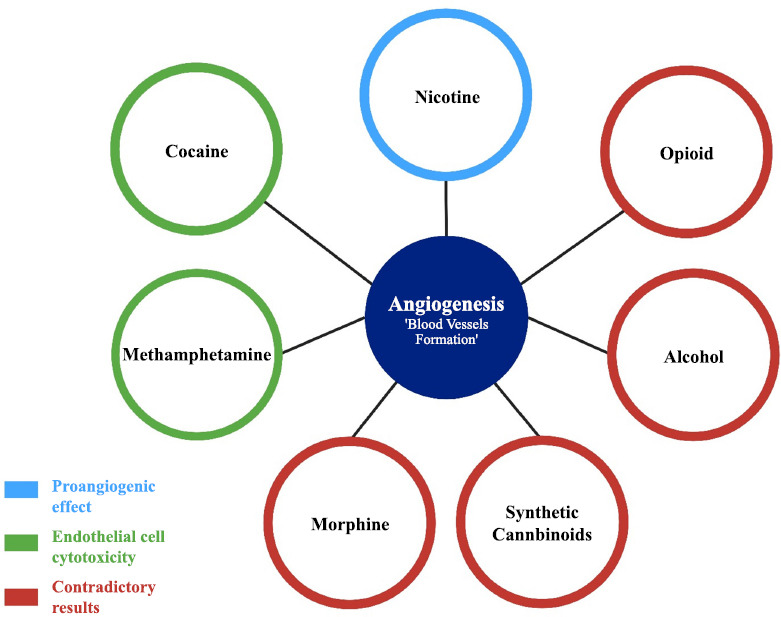
The effect of the addictive substances on the angiogenesis process. The red color refers to the substances that show contradictory results to angiogenesis, which means that proangiogenic and antiangiogenic signals. The blue color refers to the substances that show proangiogenic signals. The green color refers to the substances that cause cytotoxicity to the endothelial cells and, therefore, inhibit angiogenesis.

Addictive substances are used in medical practice and academic research. These substances may be misused by individuals, leading to a heightened risk of substance-related disorders and altered physiological and psychological functions. Several studies have shown that angiogenesis may be affected by addictive substances (Fig. **2**). While some substances may enhance angiogenesis, others may inhibit this physiological process or have little or no effect. Accordingly, many studies highlight the potential therapeutic role of these substances in the control and treatment of numerous diseases whose progression and development are enhanced through the modulation of angiogenesis. However, many challenges arise from the use of addictive substances to treat angiogenesis-related diseases, including the contradictory data resulting from various studies and the need to determine suitable dosing for effective control of angiogenesis. Further investigations are needed to elucidate the proangiogenic and antiangiogenic effects of these addictive substances. Additionally, studies should be conducted to determine appropriate dosing in order to effectively modulate the angiogenic response without producing any cytotoxic effect on the endothelial cells.

## AUTHORS’ CONTRIBUTIONS

LNA-E initiated the review. LNA-E, SZA, and IYK collected and reviewed scientific literature resources. LNA-E, SZA, and IYK wrote the draft manuscript and contributed to the final version.

## LIST OF ABBREVIATIONS

ANG: Angiopoietins
CIH: Chronic Intermittent Hypoxia
CVD: Cardiovascular Disease
ECs: Endothelial Cells
EGF: Epidermal Growth Factor
EPCs: Endothelial Progenitor Cells
FGF-2: Fibroblast Growth Factor-2
HIFs: Hypoxia-inducible Factors
HUVECs: Human Umbilical Vein Endothelial Cells
NPS: Novel Psychoactive Substances
PAI-1: Plasminogen Activator Inhibitor-1
PDGF: Platelet-derived Growth Factor
PGF: Placenta Growth Factor
rTMS: Transcranial Magnetic Stimulation
tDCS: Transcranial Direct Current Stimulation
TIMPs: Tissue Inhibitors of Metalloproteinases
TNF: Tumor Necrosis Factor
VEGF: Vascular Endothelial Growth Factors
